# A superlinear iteration method for calculation of finite length journal bearing's static equilibrium position

**DOI:** 10.1098/rsos.161059

**Published:** 2017-05-31

**Authors:** Wenjie Zhou, Xuesong Wei, Leqin Wang, Guangkuan Wu

**Affiliations:** 1 School of Energy and Power Engineering, Jiangsu University, Zhenjiang 212013, People's Republic of China; 2 Institute of Process Equipment, Zhejiang University, Hangzhou 310027, People's Republic of China; 3 Institute of Water Resources and Hydro-electric Engineering, Xi'an University of Technology, Xi'an, 710048, People's Republic of China

**Keywords:** twofold secant method, computational efficiency, journal bearing, equilibrium position, finite difference method

## Abstract

Solving the static equilibrium position is one of the most important parts of dynamic coefficients calculation and further coupled calculation of rotor system. The main contribution of this study is testing the superlinear iteration convergence method—twofold secant method, for the determination of the static equilibrium position of journal bearing with finite length. Essentially, the Reynolds equation for stable motion is solved by the finite difference method and the inner pressure is obtained by the successive over-relaxation iterative method reinforced by the compound Simpson quadrature formula. The accuracy and efficiency of the twofold secant method are higher in comparison with the secant method and dichotomy. The total number of iterative steps required for the twofold secant method are about one-third of the secant method and less than one-eighth of dichotomy for the same equilibrium position. The calculations for equilibrium position and pressure distribution for different bearing length, clearance and rotating speed were done. In the results, the eccentricity presents linear inverse proportional relationship to the attitude angle. The influence of the bearing length, clearance and bearing radius on the load-carrying capacity was also investigated. The results illustrate that larger bearing length, larger radius and smaller clearance are good for the load-carrying capacity of journal bearing. The application of the twofold secant method can greatly reduce the computational time for calculation of the dynamic coefficients and dynamic characteristics of rotor-bearing system with a journal bearing of finite length.

## 1. Introduction

A journal bearing is the main supporting component required for the stability and sustainable dynamic characteristics of the rotor system [1–5]. Many researchers are devoted to analysing the lubrication mechanism in a journal bearing. On the basis of fluids continuity equation and viscous fluid motion equation, Reynolds [6] proposed his equation, which laid the theoretical basis of fluid lubrication mechanism for journal bearing. The dynamic characteristics of journal bearing received considerable attention when Newkirk and Taylor found the unstable vibration phenomenon in journal bearing caused by the oil film [7,8]. They called this unstable phenomenon ‘oil whip’. Concurrently, Stodola regarded the fluids oil film as a simple spring support, but the model could not have explained the observed finite amplitude of oscillation of a shaft operating at a critical speed [9]. Hagg and Sankey [10,11] represented the dynamic characteristics of a journal bearing by means of two positive stiffness and two positive damping coefficients. Subsequently, Lund and Sternlicht [12–14] proposed the eight dynamic coefficients model, which improved the calculating model of a journal bearing and widely adopted in the current calculations of the rotor's dynamic characteristics (table 1).

**Table 1.** Nomenclature.

*c*	radial clearance (m)
$\bar{c}$	dimensionless radial clearance
*e*	eccentric distance (m)
*F*	lubricant film force (N)
*F*_r_, *F*_t_	radial and tangential components of dimensionless lubricant film force
${\bar{F}}_{r},\;{\bar{F}}_{t}$	dimensionless radial and tangential components of dimensionless lubricant film force
*F_w_*	external vertical load (N)
*F_x_, F_y_*	*x*- and *y*-components of lubricant film force (N)
${\bar{F}}_{x},\;{\bar{F}}_{y}$	dimensionless *x*- and *y*-components of lubricant film force
*h*	thickness of lubricant medium (m)
$\bar{h}$	dimensionless thickness
*h*_max_	maximum thickness of lubricant medium (m)
*h*_min_	minimum thickness of lubricant medium (m)
*L*	length (m)
*m*	number of grid elements in the circumferential direction
*n*	number of grid elements in the axial direction
*R*	radius (m)
*o*	geometric centre
*p*	pressure in the lubricant film (N m^−2^)
$\bar{p}$	dimensionless pressure
*t*	time (s)
$\bar{t}$	dimensionless time
*v*	velocity (m s^−1^)
*x, y, z*	axes
$\bar{x},\bar{y},\bar{z}$	dimensionless axes
$\Delta \bar{z}$	distance between two adjacent points in the axial direction
Δ*φ*	distance between two adjacent points in the circumferential direction
ε	eccentricity
*θ*	attitude angle (°)
*Λ*	length--diameter ratio
*μ*	viscosity of the lubricant medium (N s m^−2^)
*ρ*	density of the lubricant medium (kg m^−3^)
*ϕ*	acting angle (°)
*φ*	angular direction (°)
*Ω*	angular velocity (r min^−1^)
Subscript	
b	bearing
j	journal

The introduction of the dynamic characteristic coefficients was greatly convenient for the coupled solution of a rotor-bearing system. Considering the difficulty of direct solution of the analytical Reynolds equation, the short theory was applied for the engineering calculations. Alnefaie [15] researched the start-up and steady-state dynamics of a rotor supported by fluid film bearings. In this set-up, the bearing was viewed as short-plain cylindrical bearing, for which different damping ratios caused super-harmonic oscillations. Using the approximation for a short bearing, Chang-Jian and Chen [16,17] obtained the nonlinear bearing force by direct integration, which was used to facilitate the dynamic vibration of nonlinear suspension rotor. Li *et al*. [18] calculated the dynamical characteristic coefficients of a journal bearing by adopting the theory of a narrow bearing and Gümbel boundary conditions. In this research, the dynamic performance of multi-stage rotor system was also simulated for the varying dynamic coefficients and geometric parameters of journal bearings. Adiletta *et al*. [19] proposed the nonlinear dynamic model of short journal bearing under the hypothesis that the lubrication film was laminar and isothermal. The model is commonly used for a rotor system because of its good convergence and accuracy [4,20–23].

In fact, the short bearing theory was just suitable for the bearing with a small length--diameter ratio. Muszynska and Bently [24,25] proposed a simplified nonlinear fluid dynamics model that considered the circulating velocity as the key factor affecting the dynamic characteristics of a fluid film. Although the model overcame the deficiency of a short bearing compared with other bearing models, it could be adopted only for continuous fluid film, thus it is widely used to describe the nonlinear sealing force in a rotor system [26–28]. The finite difference method (FDM) [29,30], partial derivative method (PDM) [31,32] and finite element method (FEM) [33,34] are the common solving methods for eight dynamic characteristics of a journal bearing with finite length. These dynamic coefficients are important for testing the dynamic model of the rotor system with a journal bearing of finite length.

The equilibrium position is the key for solving the dynamic coefficients of a journal bearing with finite length. In order to obtain the dynamic coefficients of this bearing, the equilibrium position has to be found first [35]. Unfortunately, there are various degrees of calculation efficiency problem in the current numerical methods that determine the static equilibrium position. Therefore, in order to improve the computational efficiency of the dynamic coefficients for journal bearings of finite length and the dynamic characteristics of rotor system, in the present study, effort was made to numerically determine the equilibrium position of a journal bearing by the twofold secant method. Moreover, comparative study is done among the results of convergent iteration steps solved by the twofold secant method, secant method and dichotomy to reveal the high efficiency of the proposed superlinear iteration method. The effects of journal length, clearance and rotating speed on equilibrium position and pressure distribution were researched by the twofold secant method. Finally, analysis of the load-carrying capacity of journal bearings with different geometric parameters is also studied.

## 2. Forces in the fluids film of the journal bearing

### 2.1. The dimensionless Reynolds equation

**Figure 1.**
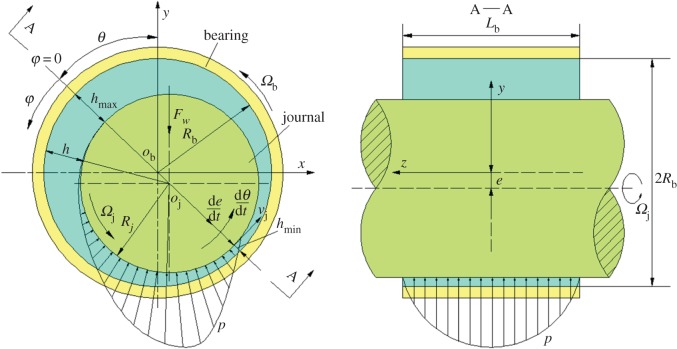
Geometric model of a journal bearing.

Figure 1 shows the geometry of a journal bearing and the coordinate system. A journal and a bearing rotate at an angular velocity of *Ω*_j_ and *Ω_b_*, respectively. The motion of the journal's centre point *o*_j_ is determined by the external vertical load and the pressure in a lubricant film.

The flow of lubricant medium between the journal and the bearing obeys the generalized Reynolds equation. In cylindrical coordinates, for cylindrical journal bearing under turbulent conditions, it reads as [36]:

2.1 $$\frac{1}{R_{b}^{2}}\frac{\partial }{\partial \varphi }\left(\frac{\rho h^{3}}{12\mu }\frac{\partial p}{\partial \varphi }\right)+\frac{\partial }{\partial z}\left(\frac{\rho h^{3}}{12\mu }\frac{\partial p}{\partial z}\right)=\frac{\left(v_{j}+v_{b}\right)}{2R_{b}}\frac{\partial (\rho h)}{\partial \varphi }+\frac{\partial (\rho h)}{\partial t}.$$

The hypothesis of iso-viscous and incompressible Newtonian lubricating liquid in a journal bearing is assumed. Considering the bearing is always fixed on a base, that is *v*_b _= *R*_b_*Ω*_b _= 0. Then, equation (2.1) can be simplified according to [34,37,38]:

2.2 $$\frac{1}{R_{b}^{2}}\frac{\partial }{\partial \varphi }\left(\frac{h^{3}}{12\mu }\frac{\partial p}{\partial \varphi }\right)+\frac{\partial }{\partial z}\left(\frac{h^{3}}{12\mu }\frac{\partial p}{\partial z}\right)=\frac{\Omega_{j}}{2}\frac{\partial h}{\partial \varphi }+\frac{\partial h}{\partial t}.$$

Introducing the following dimensionless parameters:

2.3 $$\begin{array}{rl} \bar{z} & \;=\frac{2z}{L_{b}},\;\bar{h}=\frac{h}{c}=1+\varepsilon \cos\varphi ,\\ \bar{c} & \;=\frac{c}{R_{b}},\;\bar{p}=\frac{{\bar{c}}^{2}}{6\mu \Omega_{j}}p,\;\lambda =\frac{L_{b}}{2R_{b}},\;\bar{t}=\Omega_{j}t, \end{array}$$

the dimensionless Reynolds equation can be obtained by substituting equation (2.3) in equation (2.2):

2.4 $$\frac{\partial }{\partial \varphi }\left({\bar{h}}^{3}\frac{\partial \bar{p}}{\partial \varphi }\right)+\frac{\partial }{\partial \bar{z}}\left(\frac{{\bar{h}}^{3}}{\lambda^{2}}\frac{\partial \bar{p}}{\partial \bar{z}}\right)=-\varepsilon \sin\varphi +2\varepsilon \sin\varphi \frac{\text{d}\theta }{\text{d}\bar{t}}+2\cos\varphi \frac{\text{d}\varepsilon }{\text{d}\bar{t}}.$$

As shown in figure 1, the first two items on the right-hand side of equation (2.4) represent the rotating effects of the angular velocity *Ω*_j_ and $\text{d}\theta /\text{d}\bar{t}$; the last term represents the squeezing effect of $\text{d}\varepsilon /\text{d}\bar{t}$. For the stable motion, $\text{d}\theta /\text{d}\bar{t}=\text{d}\varepsilon \text{/d}\bar{t}=0$, then equation (2.4) can be simplified into:

2.5 $$\frac{\partial }{\partial \varphi }\left({\bar{h}}^{3}\frac{\partial \bar{p}}{\partial \varphi }\right)+\frac{\partial }{\partial \bar{z}}\left(\frac{{\bar{h}}^{3}}{\lambda^{2}}\frac{\partial \bar{p}}{\partial \bar{z}}\right)=-\varepsilon \sin\varphi .$$

The boundary condition (2.6*a*) and periodic characteristic (2.6*b*) for the cylindrical journal bearing of finite length can be concluded as:

2.6a $${\bar{p}}_{\bar{z}=\pm 1}=0,\;{\frac{\partial \bar{p}}{\partial \varphi }}_{\bar{z}=0}=0$$

and

2.6b $$\bar{p}(\varphi ,\bar{z})=\bar{p}(\varphi +2\pi ,\bar{z}).$$

### 2.2. Forces in the lubricant film

**Figure 2.**
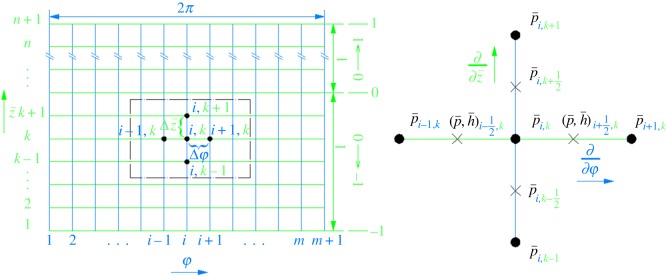
Finite difference grid.

The FDM is applied to solve the dimensionless steady-state Reynolds equation. The discretization of the film domain is usually done in two-dimensional problems [39,40]. The difference grid is shown in figure 2, where the middle point of the adjacent points is the half-integer point.

The first- and second-order derivatives and equation (2.5) are both discretized by the second-order central difference scheme. Then, equation (2.5) takes the following difference form [36]:

2.7 $$\frac{{\bar{h}}_{i+1/2}^{3}({\bar{p}}_{i+1,k}-{\bar{p}}_{i,k})-{\bar{h}}_{i-1/2}^{3}({\bar{p}}_{i,k}-{\bar{p}}_{i-1,k})}{\Delta \varphi^{2}}+\frac{{\bar{h}}_{i}^{3}}{\lambda^{2}}\frac{{\bar{p}}_{i,k+1}-2{\bar{p}}_{i,k}+{\bar{p}}_{i,k-1}}{\Delta {\bar{z}}^{2}}=-\varepsilon \sin\varphi_{i},$$

where

2.8 $$\begin{array}{rl} \Delta \varphi & \;=\frac{2\pi }{m},\;\Delta \bar{z}=\frac{2}{n},\;\varphi_{i}=(i-1)\Delta \varphi ,\;\bar{z}=(k-1)\Delta \bar{z},\\ i & \;=1,2,\ldots ,m+1,\;k=1,2,\ldots ,n+1. \end{array}$$

The resulting discretized form of dimensionless Reynolds equation takes the form:

2.9 $${\bar{p}}_{i,k}-A_{i}{\bar{p}}_{i+1,k}-B_{i}{\bar{p}}_{i-1,k}-C_{i}[{\bar{p}}_{i,k+1}+{\bar{p}}_{i,k-1}]=D_{i},$$

where

2.10 $$A_{i}=\frac{{\bar{h}}_{i+1/2}^{3}}{E_{i}\Delta \varphi^{2}},\;B_{i}=\frac{{\bar{h}}_{i-1/2}^{3}}{E_{i}\Delta \varphi^{2}},\;C_{i}=\frac{{\bar{h}}_{i}^{3}}{\lambda^{2}E_{i}\Delta {\bar{z}}^{2}},\;D_{i}=\frac{\varepsilon \sin\varphi_{i}}{E_{i}},\;E_{i}=\frac{{\bar{h}}_{i+1/2}^{3}+{\bar{h}}_{i-1/2}^{3}}{\Delta \varphi^{2}}+\frac{2{\bar{h}}_{i}^{3}}{\lambda^{2}\Delta {\bar{z}}^{2}}.$$

Analysis of equations suggest that the pressure at any grid point (*i*, *j*) is expressed in terms of pressure of four adjacent points. In order to obtain the pressure values in every discretized point quickly and accurately, equation (2.5) is solved using the successive over-relaxation iterative (SOR) method [29], supplied with the boundary conditions (2.6*a*) and periodic characteristic (2.6*b*).

**Figure 3.**
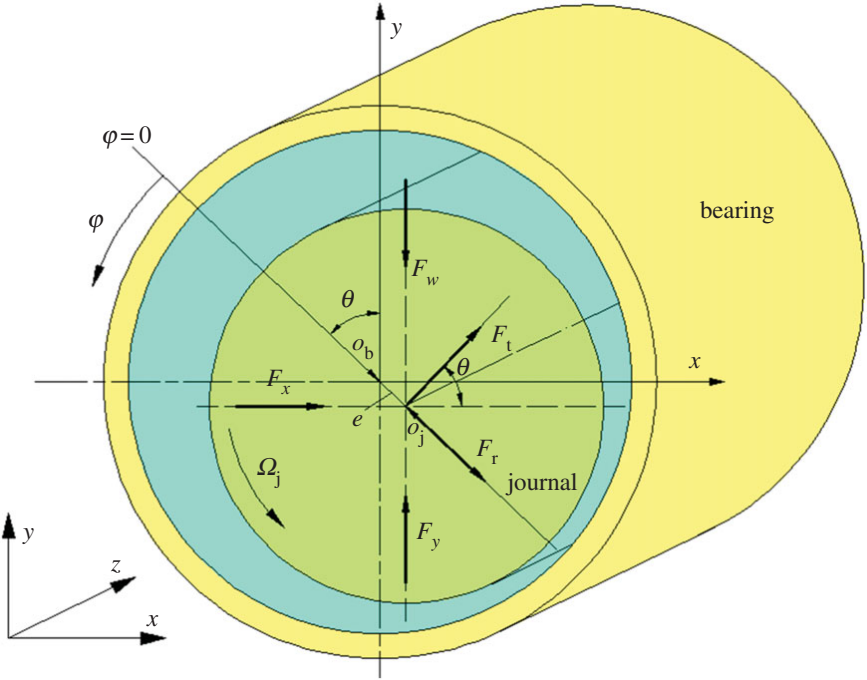
Forces induced by the lubricant film and external load.

When the lubricant film pressure distribution is calculated, components (${\bar{F}}_{t},\;{\bar{F}}_{r}$) of the dimensionless forces in the lubricant film, shown in figure 3, can be calculated using the compound Simpson quadrature formula:

2.11 $${\bar{F}}_{t}=\int_{-1}^{1}\int_{0}^{2\pi }\bar{p}\sin\varphi \text{d}\varphi \text{d}\bar{z}=\frac{\Delta \varphi \Delta \bar{z}}{9}({\bar{T}}_{1}+{\bar{T}}_{2}+{\bar{T}}_{3}+{\bar{T}}_{4})\;{\bar{F}}_{r}=\int_{-1}^{1}\int_{0}^{2\pi }\bar{p}\cos\varphi \text{d}\varphi \text{d}\bar{z}=\frac{\Delta \varphi \Delta \bar{z}}{9}({\bar{R}}_{1}+{\bar{R}}_{2}+{\bar{R}}_{3}+{\bar{R}}_{4})\}$$

where

2.12 $$\begin{array}{rl} {\bar{T}}_{1} & \;={\bar{P}}_{1}\sin\varphi_{1},\;{\bar{T}}_{2}=4\sum_{i=1}^{m/2}({\bar{P}}_{2}\sin\varphi_{2i}),\\ {\bar{T}}_{3} & \;=2\sum_{i=1}^{m/2-1}({\bar{P}}_{3}\sin\varphi_{2i+1}),\;{\bar{T}}_{4}={\bar{P}}_{4}\sin\varphi_{m+1}, \end{array}$$

2.13 $$\begin{array}{rl} {\bar{R}}_{1} & \;={\bar{P}}_{1}\cos\varphi_{1},\;{\bar{R}}_{2}=4\sum_{i=1}^{m/2}({\bar{P}}_{2}\cos\varphi_{2i}),\\ {\bar{R}}_{3} & \;=2\sum_{i=1}^{m/2-1}({\bar{P}}_{3}\cos\varphi_{2i+1}),\;{\bar{R}}_{4}={\bar{P}}_{4}\cos\varphi_{m+1} \end{array}$$

2.14 $$\begin{array}{rl} \text{and}\;{\bar{P}}_{1} & \;={\bar{p}}_{1,1}+4\sum_{k=1}^{n/2}{\bar{p}}_{1,2k}+2\sum_{k=1}^{n/2-1}{\bar{p}}_{1,2k+\text{1}}+{\bar{p}}_{1,n+1},\\ {\bar{P}}_{2} & \;={\bar{p}}_{2i,1}+4\sum_{k=1}^{n/2}{\bar{p}}_{2i,2k}+2\sum_{k=1}^{n/2-1}{\bar{p}}_{2i,2k+1}+{\bar{p}}_{2i,n+1},\\ {\bar{P}}_{3} & \;={\bar{p}}_{2i+1,1}+4\sum_{k=1}^{n/2}{\bar{p}}_{2i+1,2k}+2\sum_{k=1}^{n/2-1}{\bar{p}}_{2i+1,2k+1}+{\bar{p}}_{2i+1,n+1}\\ {\bar{P}}_{4} & \;={\bar{p}}_{m+1,1}+4\sum_{k=1}^{n/2}{\bar{p}}_{m+1,2k}+2\sum_{k=1}^{n/2-1}{\bar{p}}_{m+1,2k+1}+{\bar{p}}_{m+1,n+1}. \end{array}$$

Furthermore, the forces in the lubricant film and acting angle *ϕ* can be obtained [36]:

2.15 $$\bar{F}=\sqrt{{\bar{F}}_{t}^{2}+{\bar{F}}_{r}^{2}}$$

and

2.16 $$\phi =\left\{ \begin{array}{ll} \pi -\arcsin\left(\frac{{\bar{F}}_{t}}{\bar{F}}\right), & {\bar{F}}_{r}\geq 0\\ \arcsin\left(\frac{{\bar{F}}_{t}}{\bar{F}}\right), & {\bar{F}}_{r}<0 \end{array}\right..$$

The ${\bar{F}}_{x}$ and ${\bar{F}}_{y}$ can be calculated by transformation of the coordinates according to equation (2.11):

2.17 $${\left[\begin{array}{c} {\bar{F}}_{x}\\ {\bar{F}}_{y} \end{array}\right]}^{T}=\left[\begin{array}{cc} {\bar{F}}_{t} & {\bar{F}}_{r} \end{array}\right]\;\left[\begin{array}{cc} \cos\theta & \sin\theta \\ \sin\theta & -\cos\theta \end{array}\right].$$

## 3. Static equilibrium position

In the equilibrium state, the journal bearing of finite length must satisfy the following equilibrium condition:

3.1 $$\left\{ \begin{array}{l} F_{x}=0\\ F_{y}-F_{w}=0 \end{array}\right..$$

In other words, the equilibrium position can be determined by finding the attitude angle *θ* and eccentricity ε satisfying equation (3.1). According to the theory of fluid hydraulic lubrication, the only pressure distribution of the lubricant film can be obtained if the geometric parameters and eccentricity ε of a journal bearing used in equation (2.5) are given. The attitude angle *θ* adjusts the pressure distribution in the circumferential direction. Therefore, one must find *θ* and ε for the known external vertical load and rotating speed.

In the present work, the twofold secant method for solving nonlinear equation is applied to efficiently and accurately obtain the attitude angle *θ* and eccentricity ε. Compared to the secant method and bisection method, it has higher computational efficiency. If the initial values (${\bar{\theta }}_{0},\;\theta_{0}$) and (${\bar{\varepsilon }}_{0},\;\;\varepsilon_{0}$) are given, the iterative format of twofold secant method [41] for the static equilibrium position reads as follows:

3.2a $$\left\{ \begin{array}{l} (\text{Prediction}):{\bar{\theta }}_{i+1}=\theta_{i}-\frac{\left(\theta_{i}-{\bar{\theta }}_{i})f(\theta_{i}\right)}{f(\theta_{i})-f({\bar{\theta }}_{i})}\\ (\text{Correction}):\theta_{i+1}=\theta_{i}-\frac{\left({\bar{\theta }}_{i+1}-\theta_{i})f(\theta_{i}\right)}{f({\bar{\theta }}_{i+1})-f(\theta_{i})} \end{array}\right.\;i=0,1,2,\ldots$$

and

3.2b $$\left\{ \begin{array}{l} (\text{Prediction}):{\bar{\varepsilon }}_{j+1}=\varepsilon_{j}-\frac{\left(\varepsilon_{j}-{\bar{\varepsilon }}_{j})g(\varepsilon_{j}\right)}{g(\varepsilon_{j})-g(\bar{\varepsilon })}\\ (\text{Correction}):\varepsilon_{j+1}=\varepsilon_{j}-\frac{\left({\bar{\varepsilon }}_{j+1}-\varepsilon_{j})g(\varepsilon_{j}\right)}{g({\bar{\varepsilon }}_{j+1})-g(\varepsilon_{j})} \end{array}\right.\;j=0,1,2,\cdots .$$

The convergence criteria of equation (3.2*a*) [42] and equation (3.2*b*) give:

3.3a $$f(\theta )=\left|\frac{F_{x}}{F_{y}}\right|<10^{-3}$$

and

3.3b $$g(\varepsilon )=\left|\frac{F_{y}-F_{w}}{F_{w}}\right|<10^{-3}.$$

**Figure 4.**
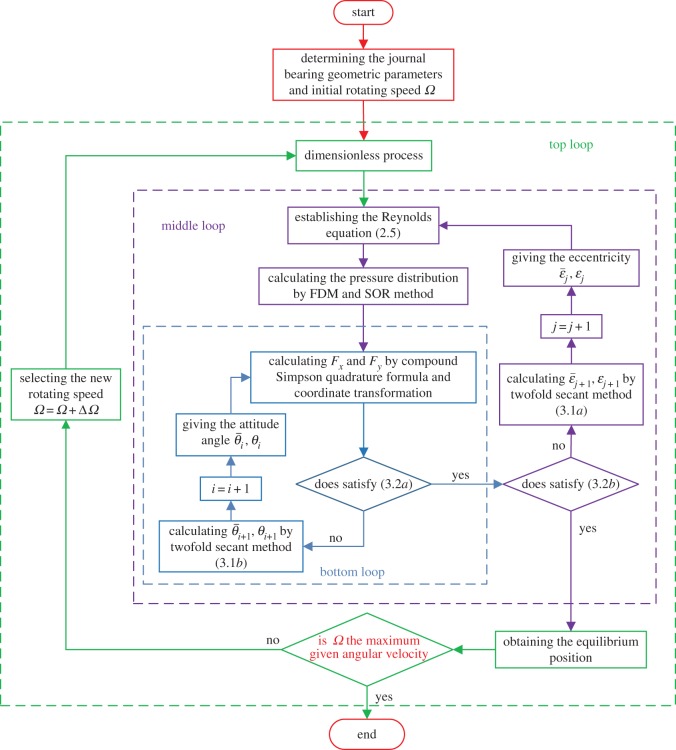
Algorithm of solution for the equilibrium position.

There are two independent variables in the iterative process; therefore, it is impossible to acquire the attitude angle and eccentricity simultaneously. Thus, the iterative process of attitude angle is embedded in the iterative process for eccentricity. Moreover, the equilibrium position depends on the rotating speed [15], i.e. it changes for the different rotating speed. Therefore, the equilibrium position can be determined for certain rotating speed and geometric parameters. The specific iterative process for static equilibrium position for the different rotating speed is shown in figure 4.

## 4. Results and discussion

The static equilibrium position of a journal bearing was obtained by the twofold secant method. The used calculating parameters of the journal bearing are shown in table 2 (figure 1 for reference). The convergence process, the inner pressure distribution and the load-carrying capacity were presented using the superlinear iteration method. The influence of the geometric parameters and the working conditions on the characteristics of journal bearing were also analysed.

**Table 2.** Calculating parameters of the journal bearing.

parameters	geometric parameters			physical parameters			grid parameters	
symbol	*c*, mm	*L*_b_, m	*R*_b_, m	*F_w_*, N	*Ω*_j_, r min^−1^	*μ*, N s m^−2^	*m*	*n*
value	0.6	0.12	0.1	1962	3000	0.04	100	20

### 4.1. Comparison of three convergence methods

In this section, in order to present the advantage of the twofold secant method for identification of the static equilibrium position, the convergence process and iterative steps of three methods are shown for different length *L*_b_, radial clearance *c* and rotating speed *Ω*_j_. Considering the accuracy and efficiency of the dichotomy and the scant method for nonlinear equations [42–44], these two methods were selected for calculation of the equilibrium position, and the results were compared with the results obtained by the twofold secant method.

**Figure 5.**
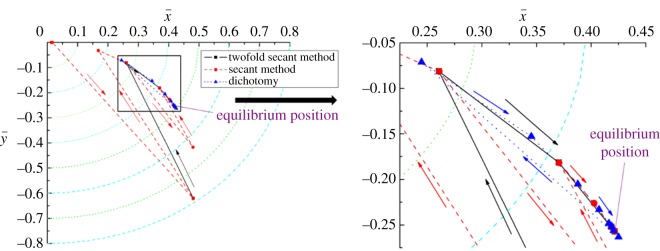
Convergence for the equilibrium position, *L*_b_ = 0.1 m.

Figure 5 presents the process of identification of the journal's equilibrium position when journal length was set to 0.1 m. The other parameters of the journal bearing are listed in table 2. It can be seen that the convergence curve of the secant method is most twisty and its iterative range is also largest compared with those of twofold secant and dichotomy methods. There is only one fold point for the twofold secant method and the dichotomy method in the seeking process. Thus, these two methods effectively reduce the seeking scope at the beginning of the iteration process. However, for the dichotomy approach, the high density of the points shows poor convergence performance near the equilibrium position. Moreover, all three curves converged to the same equilibrium position point (ε = 0.489, *θ* = 59°). As this figure shows, the calculating efficiency of the twofold secant method is superior to the secant method and dichotomy.

**Figure 6.**
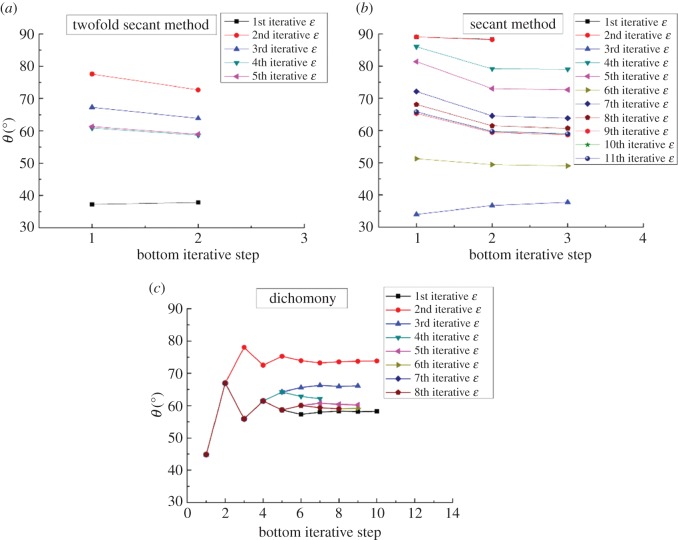
(*a*–*c*) Convergence for the attitude angle, *L*_b_ = 0.1 m.

Figure 6 represents the convergence process of attitude angle corresponding to the different iterative number of eccentricity shown in figure 5. It can be seen from figure 6*a–c* that the iterative steps of attitude angle, i.e. the number of iterations in bottom loop, increase from the twofold secant method to dichotomy. The maximum numbers of iterative steps of the twofold secant method, secant method and dichotomy are 2, 3 10, respectively. Therefore, the convergence efficiency for attitude angle decreases from the twofold secant method to dichotomy. Also, compared with the iterative process of middle loop and bottom loop using the secant method, the numbers of iterations for attitude angle are much less than those for eccentricity. This is because the convergence efficiency for the secant method is mainly affected by the monotonicity of iterative results.

**Figure 7.**
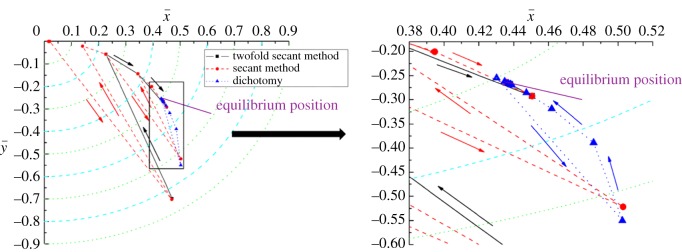
Convergence for the equilibrium position, *c *= 0.8 mm.

**Figure 8.**
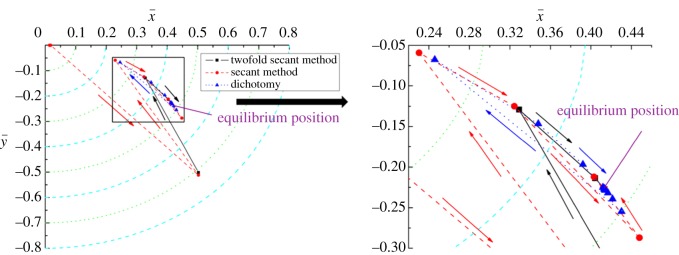
Convergence for the equilibrium position, *Ω*_j_ = 2000 r min^−1^.

The convergence process for the equilibrium position when the clearance is 0.8 mm and the rotating speed is 2000 r min^−1^ is plotted in figures 7 and 8, respectively. From the two figures, it is clearly shown that the convergence trajectory for all three methods is nearly the same. The convergence curve of the secant method has more folds than the other two curves, and the convergence efficiency of the twofold secant method is also higher than that of the secant method and dichotomy. Besides, for the dichotomy, when the iterative values approach the point of convergence, the convergence speed becomes slow sharply. This accounts for the low convergence efficiency of the dichotomy for equilibrium position compared with the other two methods.

Table 3 shows the iterative steps of bottom loop and middle loop for different bearing length, clearance and rotating speed. As indicated in table 3, there are less iterative steps for the twofold secant method than those for the secant method and dichotomy. The final equilibrium positions obtained by each iterative method for each set of conditions are nearly the same. The tiny discrepancy can be attributed to the different values of convergence condition (equations (3.3*a*) and (3.3*b*)).

**Table 3.** Iterative steps of bottom loop and middle loop.

	maximum iterative steps for ε (middle loop)			maximum iterative steps for *θ* (bottom loop)		
method	*L*_b_ = 0.1 m	*c *= 0.8 mm	*Ω*_j_ = 2000 r min^−1^	*L*_b_ = 0.1 m	*c *= 0.8 mm	*Ω*_j_ = 2000 r min^−1^
twofold secant	5	6	4	2	2	2
secant	11	12	9	3	3	3
dichotomy	8	9	12	10	10	10

	equilibrium position: ε, *θ*					
	method	*L*_b _= 0.1 m	*c *= 0.8 mm		*Ω*_j_ = 2000 r min^−1^	
twofold secant	0.4886, 58.952°		0.5133, 58.558°		0.4721, 61.191°	
secant	0.4886, 58.956°		0.5133, 58.562°		0.4721, 61.195°	
dichotomy	0.4885, 59.015°		0.5134, 58.582°		0.4720, 61.176°	

The computational time of three different methods for different calculating parameters is listed in table 4. The computation time of the twofold secant method is significantly less than that of the secant method and dichotomy. However, there are no obvious advantages and disadvantages to the secant method and dichotomy. The comparative results, obtained from tables 3 and 4, clearly show that the computational efficiency of the twofold secant method is obviously higher than that of the two traditional iteration methods.

**Table 4.** The computational time of three different methods.

	computational time×10^2 ^s			
method	initial parameters	*L*_b_ = 0.1 m	*c *= 0.8 mm	*Ω*_j_ = 2000 r min^−1^
twofold secant	0.131363	0.218753	0.231381	0.184579
secant	0.189367	0.275931	0.283670	0.240107
dichotomy	0.294895	0.223008	0.271227	0.277970

### 4.2 The effects of *L*_b_, *c* and *Ω*_j_ on the equilibrium position

The numerical results calculated by the twofold secant method are compared with those of previous reference [45]. The comparative results are listed in table 5. It is evident through this table that the equilibrium position and lubrication film force calculated by the superlinear iteration method are consistent with those of previous reference. The maximum relative error and minimum relative error are 3.14% and 0.06%, respectively. The small difference of the numerical results implies that the twofold secant method proposed in the paper is accurate and feasible.

**Table 5.** Comparison of the numerical results.

parameter	results of ref. [45]	results of the twofold secant method	relative error (%)
eccentricity ε	0.3932	0.3989	1.45
attitude angle *θ*, (°)	63.7	65.7	3.14
lubricant film force |*F_y_*|, N	60.005	59.967	0.06

In addition, in order to investigate the influence of geometric parameters and working condition on the equilibrium position, the bearing length *L*_b_, clearance *c* and rotating speed *Ω*_j_ were selected as the independent variables to be used in further calculations by the twofold secant method.

**Figure 9.**
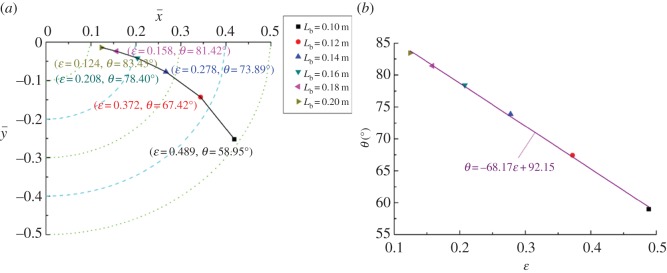
Influence of *L*_b_ on the equilibrium position (*a*) and the corresponding fitting curve (*b*).

The parameters used in the calculation of different equilibrium positions, shown in figure 9*a*, are listed in table 2 without the bearing length *L*_b_. It can be seen that the equilibrium position gradually approaches the geometric centre of the bearing when the length increases from 0.1 to 0.2 m. This reflects on the eccentricity and attitude angle—ε decreases from 0.489 to 0.124, while *θ* increases from 58.95° to 83.43°. This phenomenon illustrates that higher bearing length facilitates the load-carrying capacity of the journal bearing. This fact is consistent with the results of Gengyuan *et al*. [46].

The corresponding fitting curve of the eccentricity and the attitude angle is plotted on figure 9*b*. The straight line, fitted by the least square method, indicates that the relationship of ε and *θ* is linear to the change of the bearing length.

**Figure 10.**
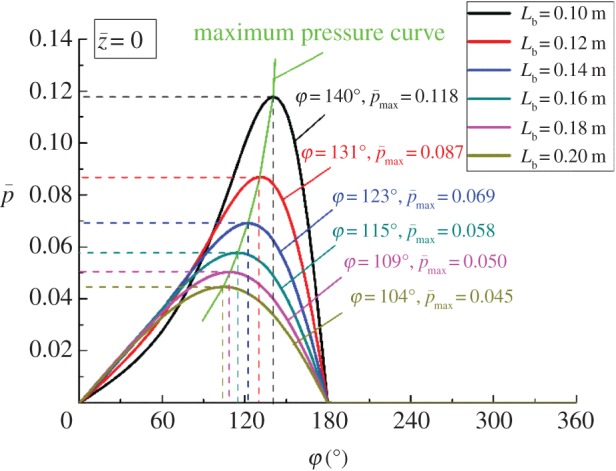
Pressure distribution with different *L*_b_ at $\bar{z}=0$.

**Figure 11.**
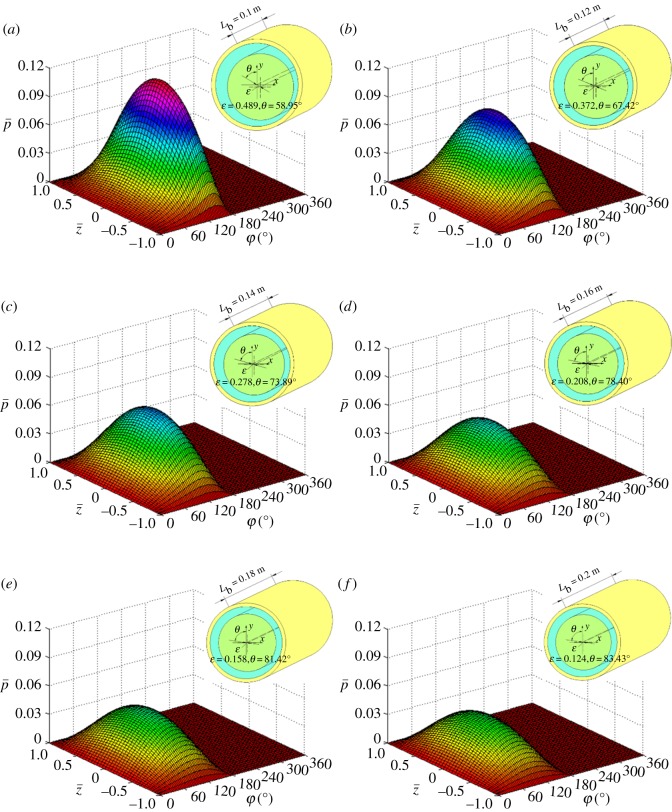
Pressure distribution of whole flow field with different *L*_b._

Figure 10 presents the distribution curves of the dimensionless pressure for corresponding equilibrium positions shown in figure 9 ($\bar{z}=0$). Figure 10 obviously shows that the peak pressure decreases as bearing length increases and the maximum dimensionless pressure of 0.118 is achieved when *L*_b _= 0.1 m. The reason for this result is certain equilibrium position. The smaller *L*_b_ with larger eccentricity can decrease the minimum flow clearance, and this means that a bearing with smaller *L*_b_ can reduce pressure leakage and hold the pressure better than a bearing with larger *L*_b_. Moreover, the angular position of the maximum pressure changes from 140° to 104° when *L*_b_ increases from 0.1 to 0.2 m. That is, for smaller *L*_b_, the equilibrium position of maximum pressure area is closer to the *h*_min_ (figure 11).

The pressure distribution in the entire flow field clearly shows that the positive pressure emerges in the convergent wedge (*φ* = 0° → 180°). Theoretically, once the lubrication fluid crosses the minimum clearance and enters the divergence wedge (*φ* = 180° → 360°), the pressure quickly decreases and the values change from positive to negative on account of the expansion effect.

In fact, the actual lubrication fluid cannot bear the tensile stress and the liquid film will fracture in the divergence wedge, this is why half Sommerfeld boundary condition was widely applied in the calculations of Reynolds equation [38,47–49]. In addition, the pressure is distributed symmetrically on the left and right side of central plane ($\bar{z}=0$) in the axial direction.

**Figure 12.**
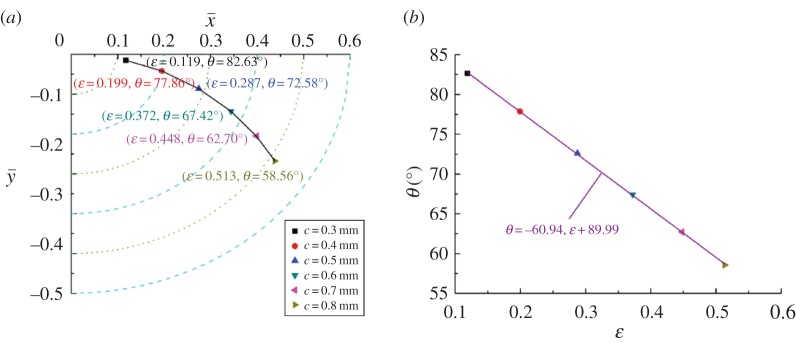
Influence of *c* on the equilibrium position (*a*) and corresponding fitting curve (*b*).

**Figure 13.**
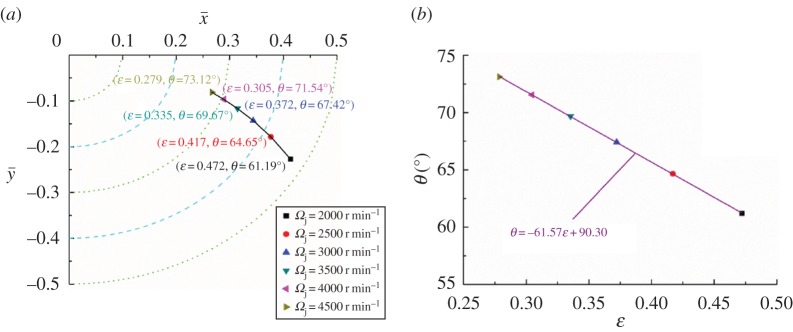
Influence of *Ω*_j_ on the equilibrium position (*a*) and corresponding fitting curve (*b*).

The changing trajectory of equilibrium positions obtained by the twofold secant method and the corresponding fitting curve for different clearances and rotating speeds are shown in figures 12 and 13, respectively. Similar to figure 9*a*, the changing trajectory presents parabolic characteristics. The difference in the three trajectory curves could be explained by the fact that the equilibrium position is more sensitive for both bearing length and clearance, and less sensitive for rotating speed. This is shown in the changing range of equilibrium positions: in figures 9*a* and 12*a* it is larger than that shown in figure 13*a*. It also can be seen that with the increase of clearance, the eccentricity increases from 0.119 to 0.513. By contrast, the eccentricity decreases from 0.472 to 0.279 as the rotating speed changes from 2000 to 4500 r min^−1^. The calculated results imply that smaller clearance and higher rotating speed is beneficial to load-carrying capacity.

Figures 12*b* and 13*b*, respectively, present the relationship between the eccentricity and the attitude angle for different clearances and rotating speeds. Similarly, the negative slope of the two fitting curves demonstrates that eccentricity and attitude angle are in the inverse proportional relationship. Whereas compared with the slope of the fitting curve for bearing length shown in figure 9*b*, the slopes of clearance and rotating speed change from −68.17 to −60.94 and −61.57, respectively. These findings mean that the variation of attitude angle caused by the bearing length is greater than that of the clearance or the rotating speed for the same variation of the eccentricity.

**Figure 14.**
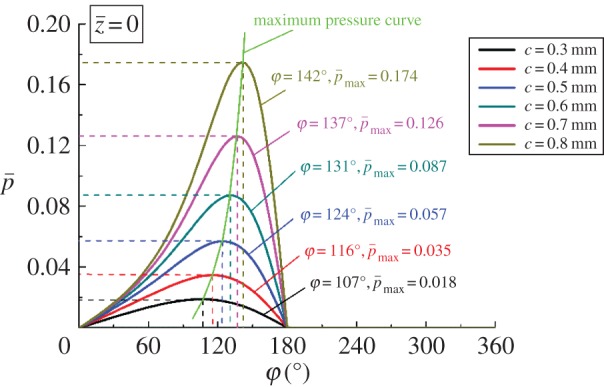
Pressure distribution with different *c* at $\bar{z}=0$.

**Figure 15.**
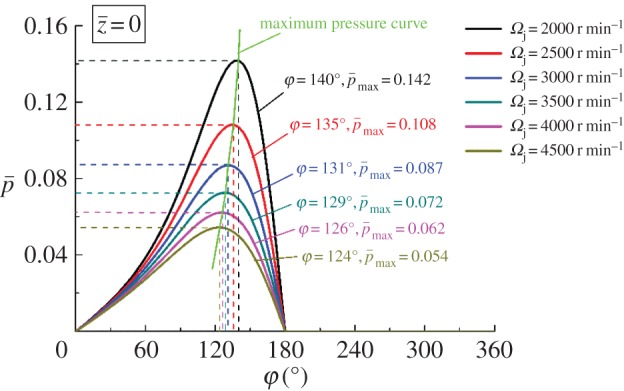
Pressure distribution with different *Ω*_j_ at $\bar{z}=0$.

The pressure distribution curves corresponding to the equilibrium positions shown in figures 12*a* and 13*a* are obtained to describe the variation of pressure in axial direction on the central plane. According to figures 14 and 15, the maximum pressure generates when *c *= 0.8 mm and *Ω*_j_ = 2000 r min^−1^. Combining these findings with the conclusion obtained from figure 10, it is clear that the larger eccentricity can cause higher pressure under the same geometric parameters and working condition. The angular position of the maximum pressure decreases with reduction of the clearance or rise of the rotating speed. Moreover, the increments for the maximum pressure and angle position increase as the bearing length decreases or the clearance increases, as shown in figures 10 and 14, respectively.

### 4.3. The effects of *L*_b_, *c* and *R*_b_ on the forces in lubricant film

The geometric parameters, including *L*_b_, *c* and *R*_b_, play an important role in lubricant film force, directly affect the load-carrying capacity and the equilibrium position of the journal bearing and finally determine the calculated dynamic coefficients. In order to determine the equilibrium position using the proposed model, the lubricant film force needs to be transformed into dimensionless form [47,50]. In this section, the main geometric parameters *L*_b_, *c* and *R*_b_ are taken into consideration for investigation of the lubricant film forces for different eccentricities.

**Figure 16.**
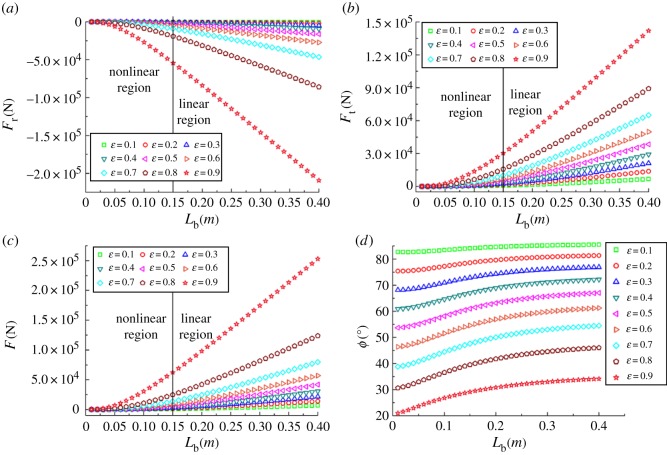
Lubricant film forces and the acting angles.

Figure 16*a*–*c* presents the changing trend for the radial film force *F*_r_, the tangential film force *F*_t_ and the total lubricant film force *F*, respectively, for the different bearing lengths and eccentricity changing from 0.1 to 0.9. The negative values of *F*_r_ illustrate that the actual acting direction of the radial film force is opposite to the direction shown in figure 3. The rising curves given in figure 16*a*–*c* also demonstrate that when the eccentricity is fixed, the values of film force with larger *L*_b_ are obviously greater than those with smaller *L*_b_; the same results are also true for the fixed *L*_b_ and changing eccentricity. That is, larger bearing length and eccentricity have a positive role in improving the load-carrying capacity of journal bearing. In addition, the curves of the film's forces can be roughly divided into nonlinear region and linear region at *L*_b_ = 0.15 m on the basis of the slope change. The relationship of the acting angle and the bearing length with the different eccentricities is shown in figure 16*d*. Actually, the changing trend of the acting angle reflects the *F*_t_–*F* ratio according to the equation (2.16). As mentioned in figure 16*d*, the curve becomes more flat for smaller eccentricity; the values of acting angle with larger bearing length are greater than those of smaller bearing length for the fixed eccentricity.

It can be seen in table 6 that the changing trend of lubricant film force with fixed eccentricity satisfies the quadratic polynomial growth for clearance ranging from 1.0 to 0.1 mm. Also, the quadratic changing trend can be found when the clearance is fixed, and the eccentricity increases from 0.1 to 0.9. The phenomenon is ascribed to the quadratic term of eccentricity ε in dimensional conversion. Therefore, decreasing the clearance or increasing the eccentricity is a good way of improving the load-carrying capacity of a journal bearing. However, when the clearance is reduced to a certain degree, the rubbing phenomenon emerges and seriously affects the stability of rotor-bearing system [26]. That is, the choice of clearance needs to take the combined effect of various factors into consideration.

**Table 6.** Lubricant film force *F* (N) for different clearances and eccentricities.

	lubricant film force *F* (N) for eccentricity ε								
radial clearance *c*, mm	ε = 0.1	0.2	0.3	0.4	0.5	0.6	0.7	0.8	0.9
*c *= 0.1	14 380	31 175	51 607	78 828	118 533	182 654	300 944	570 322	1 528 820
0.2	3595	7794	12 902	19 707	29 633	45 664	75 236	142 580	382 205
0.3	1598	3464	5734	8759	13 170	20 295	33 438	63 369	169 869
0.4	899	1948	3225	4927	7408	11 416	18 809	35 645	95 551
0.5	575	1247	2064	3153	4741	7306	12 038	22 813	61 153
0.6	399	866	1434	2190	3293	5074	8360	15 842	42 467
0.7	293	636	1053	1609	2419	3728	6142	11 639	31 200
0.8	225	487	806	1232	1852	2854	4702	8911	23 888
0.9	178	385	637	973	1463	2255	3715	7041	18 874
1.0	144	312	516	788	1185	1827	3009	5703	15 288

The aim of table 7 is to present the relationship between the bearing radius and the lubricant film force under different eccentricities. In this case, the forces grow with fixed eccentricity when the bearing radius increases from 0.05 to 0.15 m. This fact means that the journal bearing with larger radius (*R*_b_ = 0.15 m) has better load-carrying capacity compared with those of smaller radius (*R*_b_ = 0.05 m).

**Table 7.** Lubricant film force *F* (N) for different bearing radii and eccentricities.

	lubricant film force *F* (N) for eccentricity ε								
radius of bearing *R*_b_, m	ε = 0.1	0.2	0.3	0.4	0.5	0.6	0.7	0.8	0.9
*R*_b_ = 0.05	149	312	497	722	1020	1451	2159	3584	7999
0.06	201	423	680	1001	1436	2085	3182	5456	12 698
0.07	252	535	867	1292	1880	2779	4339	7661	18 549
0.08	303	647	1057	1589	2341	3516	5603	10 155	25 508
0.09	352	757	1246	1889	2814	4284	6949	12 896	33 507
0.10	399	866	1434	2190	3293	5074	8360	15 842	42 467
0.11	445	973	1620	2490	3775	5878	9818	18 959	52 305
0.12	489	1078	1803	2789	4259	6692	11 313	22 216	62 932
0.13	531	1180	1985	3086	4742	7512	12 834	25 586	74 266
0.14	572	1280	2164	3381	5224	8334	14 374	29 047	86 228
0.15	611	1378	2341	3674	5705	9158	15 927	32 580	98 742

## 5. Conclusion

A novel superlinear iteration convergence method—twofold secant method, was applied for identification of the equilibrium position of journal bearing of finite length. The Reynolds equation for stable motion was solved numerically and effects of the geometric parameters and the operating condition on equilibrium position, pressure distribution and load-carrying capacity were studied. Main conclusions are as follows:

(1) The number of iterative steps of the proposed method—twofold secant method---required for obtaining equilibrium position are obviously less than for the present methods: secant method and dichotomy. The efficiency of calculation of dynamic coefficients and dynamic characteristics of journal bearing with finite length using the twofold secant method is faster.
(2) The trajectory of equilibrium positions is parabolic and the relationship curves between attitude angle *θ* and eccentricity ε fitted by the least square method indicates that the attitude angle linearly decreases when the eccentricity increases. In addition, a variation of the attitude angle is more sensitive to the bearing length than to the clearance or the rotating speed.
(3) The inner pressure is distributed symmetrically in the axial direction on the left and right side relative to the central plane ($\bar{z}=0$). The large bearing length (*L*_b_ = 0.2 m), higher rotating speed (*Ω*_j_ = 3500 r min^−1^) and small clearance (*c* = 0.3 mm) shift the angle position of maximum pressure away from the position of minimum clearance between journal and bearing for the same external vertical loads.
(4) The geometric parameters, including *L*_b_, *c* and *R*_b_, have a major impact on the load-carrying capacity. The load-carrying capacity can be enhanced by enlarging the bearing length, its radius or reducing the clearance. Besides, the curves of load-carrying capacity can be divided into nonlinear region and linear region according to the slopes as the eccentricity increases from 0.1 to 0.9.

## Data accessibility

Data are available from the Dryad Digital Repository: (http://dx.doi.org/10.5061/dryad.6n0m8) [51].
